# Haploinsufficiency and the sex chromosomes from yeasts to humans

**DOI:** 10.1186/1741-7007-9-15

**Published:** 2011-02-28

**Authors:** Michaela de Clare, Pınar Pir, Stephen G Oliver

**Affiliations:** 1 Cambridge Systems Biology Centre and Department of Biochemistry, University of Cambridge, Sanger Building, 80 Tennis Court Road, Cambridge CB2 1GA, UK

## Abstract

**Background:**

Haploinsufficient (HI) genes are those for which a reduction in copy number in a diploid from two to one results in significantly reduced fitness. Haploinsufficiency is increasingly implicated in human disease, and so predicting this phenotype could provide insights into the genetic mechanisms behind many human diseases, including some cancers.

**Results:**

In the present work we show that orthologues of *Saccharomyces cerevisiae *HI genes are preferentially retained across the kingdom Fungi, and that the HI genes of *S. cerevisiae *can be used to predict haploinsufficiency in humans. Our HI gene predictions confirm known associations between haploinsufficiency and genetic disease, and predict several further disorders in which the phenotype may be relevant. Haploinsufficiency is also clearly relevant to the gene-dosage imbalances inherent in eukaryotic sex-determination systems. In *S. cerevisiae*, HI genes are over-represented on chromosome III, the chromosome that determines yeast's mating type. This may be a device to select against the loss of one copy of chromosome III from a diploid. We found that orthologues of *S. cerevisiae *HI genes are also over-represented on the mating-type chromosomes of other yeasts and filamentous fungi. In animals with heterogametic sex determination, accumulation of HI genes on the sex chromosomes would compromise fitness in both sexes, given X chromosome inactivation in females. We found that orthologues of *S. cerevisiae *HI genes are significantly under-represented on the X chromosomes of mammals and of *Caenorhabditis elegans*. There is no X inactivation in *Drosophila melanogaster *(increased expression of X in the male is used instead) and, in this species, we found no depletion of orthologues to yeast HI genes on the sex chromosomes.

**Conclusion:**

A special relationship between HI genes and the sex/mating-type chromosome extends from *S. cerevisiae *to *Homo sapiens*, with the microbe being a useful model for species throughout the evolutionary range. Furthermore, haploinsufficiency in yeast can predict the phenotype in higher organisms.

## Background

The relevance of haploinsufficiency (a deleterious phenotype upon the reduction of gene copy number from two to one in a diploid cell) to human disease is being increasingly appreciated. Haploinsufficiency for over 20 tumour-suppressor genes (including the *PTEN, BRCA1 *and *2*, and *p53 *[1], and *RUNX *genes [2]) has been demonstrated to contribute to tumourigenesis and metastasis. It is also implicated in inherited conditions such as Turner syndrome [3]; Stickler syndrome [4]; ribosome biogenesis disorders [5], and other developmental abnormalities [3].

Although several of these disease associations have been recently confirmed with the construction of heterozygous deletion mouse mutants [6], performing large-scale *in vivo *screens for haploinsufficient (HI) genes in the higher eukaryotes is still hampered by the inherent difficulties both in constructing deletion mutants and quantifying the resulting phenotype. By contrast, for the model unicellular eukaryote *Saccharomyces cerevisiae*, there exists a library of diploid strains each carrying a deletion of one copy of an individual protein-coding gene in the genome [7], and growth can be easily quantified either in batch or continuous culture. Since each strain within this heterozygous deletion library is constructed incorporating a unique 20-mer 'barcode' [7], mutant strains representing the entire genome can be grown in a single competitive pool [8].

Using this approach, we previously defined sets of haploinsufficient genes in *Saccharomyces cerevisiae *under several different nutrient conditions by competition experiments [8]. More recently, we defined a set of approximately 1,000 HI genes growing in rich medium in a fed-on-demand turbidostat culture (P Pir, A Gutteridge, J Wu, B Rash, DB Kell, N Zhang, SG Oliver, unpublished). In such cultures, a feedback loop, varying the input rate of fresh growth medium, acts to maintain a constant cell density. Thus, population growth can be maintained at the highest possible rate [9,10]. These conditions allow us to identify those genes which are phenotype is independent of any specific nutrient limitation. It is this set of HI genes upon which we have concentrated in the present work.

The phenotypes of yeast strains carrying mutations for conserved genes have been long established as being highly predictive for human genetic disorders [11]; that is, the function of a human protein, and its contribution to a particular phenotype, can be inferred from that of its yeast orthologue [12]. This 'phenologue' approach is being increasingly justified as confirmation experiments are performed in more complex higher organisms [13] and conditions including ageing, cancer, neurodegenerative and prion disease, apoptosis, and DNA repair disorders are studied in yeast [12]. In addition, yeast screens are widely used to identify gene targets for potential therapeutic intervention [14].

We therefore sought to apply such an orthology-based approach to relate haploinsufficiency in yeast and humans, particularly with respect to known disease genes, by examining the evolutionary significance of the *S. cerevisiae *HI genes, their conservation across species, and the degree to which yeast haploinsufficiency can predict the phenotype in higher organisms.

## Results

### HI genes are preferentially retained across the eukaryotes

By examining orthology relationships, we found that orthologues of *S. cerevisiae *HI genes are highly retained among the yeast species; the probability of their having an orthologue in another yeast species is significantly higher than the average for the *S. cerevisiae *genome (Fisher's exact test; *P *= 10^-3^). We found that this is true with respect to both those species which diverged prior to, and those diverging after, the whole-genome duplication (WGD) that occurred approximately 100 million years ago [15] in the Saccharomycetales lineage. Moreover, HI genes are also more likely to have subsequently been retained in duplicate following the WGD (see *P *values below), indicating that, at least through the approximately 20 million year period of rapid post-WGD gene shedding (between the divergence of the *S. cerevisiae *and *Kluveromyces polysporus *lineages [16]), selective pressure existed toward maintaining a higher dosage of the proteins they encode.

Since ribosomal complexes are acutely sensitive to protein dosage imbalance, and most genes encoding ribosomal proteins (r-proteins) are over-represented among the gene pairs retained following the WGD [17], we excluded r-protein genes from the analysis and still found the *S. cerevisiae *genome to be enriched for pairs containing at least one (*P *< 10^-5^), or two (*P *= 0.007) HI members (see Additional file 1). A similar pattern is observed in other post-WGD yeast species, with orthologues of HI genes being over-represented among surviving WGD paralogous pairs (*P = *10^-7^, 0.05, 0.01 for *Saccharomyces bayanus*, *K. polysporus *and *Saccharomyces castellii*, respectively). The *K. polysporus *result is particularly significant, since post-WGD gene loss in this species occurred essentially independently of *S. cerevisiae *evolution [16]. All of this suggests that the haploinsufficient phenotype predated the WGD.

The retention of orthologues of yeast HI genes appears to have acted over a still greater evolutionary timeframe: we found that yeast HI orthologues are highly retained across the entire Ascomycete phylum, an evolutionary span of at least 900 million years [18], and that the orthologues of yeast HI genes are retained significantly more frequently than the genomic average in 75% of eukaryotic species with published sequenced genomes (see *P *values in Tables 1 and 2). We considered the possibility that this over-representation is driven by a subset of the HI genes coding for proteins involved in some important biological process that is, itself, highly conserved among eukaryotes. In particular, the set of HI genes in *S. cerevisiae *is enriched for genes encoding r-proteins. In order to rule out this possibility, the HI genes were first classified into the broad functional categories represented by the generic *S. cerevisiae *GO_slim terms provided by the Gene Ontology Consortium [19]. Genes within each of these discrete sets were then sequentially removed and the orthology analysis repeated. In each case, the statistical significances of the orthology comparisons remained at *P *< 0.05; for those species in which the full HI set is more highly retained, the significant over-representation is preserved and, for those few species not exhibiting HI-orthologue over-representation, deletion of functional categories does not give rise to significance. This suggests that the over-representation of orthologues is indeed due to haploinsufficiency, rather than any particular functional subset.

**Table 1:** Phylogenetic relationships of the Ascomycete fungi

Class	Species	*P *value of increased HI orthologue retention
Saccharomycetes:		

Post-WGD yeasts	*Saccharomyces cerevisiae*	-

	*Saccharomyces paradoxus*	0.002

	*Saccharomyces bayanus*	0.001

	*Kluyveromyces polysporus*	0.01

	*Saccharomyces castellii*	0.009

Pre-WGD yeasts	*Saccharomyces kluyveri*	0.04

	*Kluyveromyces waltii*	0.004

	*Ashbya gossypii*	0.003

	*Kluyveromyces lactis*	0.001

	*Candida albicans*	0.01

	*Candida tropicalis*	0.001

	*Candida parapsilosis*	0.0004

	*Lodderomyces elongisporus*	0.0006

	*Candida lusitaniae*	0.001

	*Debaryomyces hansenii*	0.006

	*Candida guilliermondii*	0.001

	*Yarrowia lipolytica*	0.02

Eurotiomycetes	*Aspergillus nidulans*	0.0009

Sordariomycetes	*Neurospora crassa*	0.0005

	*Magnaporthe grisea*	0.03

Schizosaccharomycetes	*Schizosaccharomyces pombe*	0.01

	*Schizosaccharomyces japonicus*	0.001

The *P *value for *Saccharomyces cerevisiae *haploinsufficient (HI) genes having a greater likelihood of having an orthologue, relative to the genome average, is shown for each species. Ribosomal protein genes were excluded from the analysis (see main text).

WGD = whole-genome duplication.

**Table 2:** Phylogenetic relationships of representative model eukaryotes

Kingdom	Phylum	Species	*P *value of increased HI orthologue retention
Chromalveolata		*Giardia lamblia*	0.2

		*Leishmania major*	0.1

		*Thalassiosira pseudonana*	0.1

		*Cryptosporidium hominus*	0.8

		*Plasmodium falciparum*	0.2

Planta		*Oryza sativa*	0.05

		*Arabidopsis thaliana*	0.02

Amoebazoa		*Dictyostelium discoideum*	0.2

Fungi		*Schizosaccharomyces pombe*	0.01

		*Ashyba gossypii*	0.003

		*Saccharomyces cerevisiae*	

Animalia	Nematodes	*Caenorhabditis elegans*	0.05

		*Caenorhabditis briggsae*	0.05

	Insects	*Drosophila melanogaster*	0.05

		*Anopheles gambiae*	0.01

	Fish	*Takifugu rubripes*	0.01

		*Tetraodon nigroviridis*	0.04

		*Danio rerio*	0.01

	Birds	*Gallus gallus*	0.2

	Mammals	*Mus musculus*	0.01

		*Rattus norvegicus*	0.05

		*Equus caballus*	0.05

		*Bos taurus*	0.03

		*Canis familiaris*	0.04

		*Homo sapiens*	0.04

		*Pan troglodytes*	0.04

The *P *value for *Saccharomyces cerevisiae *haploinsufficient (HI) genes having a greater likelihood of having an orthologue, relative to the genome average, is shown for each species.

Similarly, we also sought to rule out the possibility that conservation is driven by the *homozygous *deletant phenotype, rather than the HI phenotype. Accordingly, we first confirmed that, congruent with our previous findings for chemostat culture [8], there exists no significant correlation between our turbidostat haploinsufficient set and essentiality/significant fitness defect in the homozygous deletant (*P *value of an overlap between the phenotypes = 0.7). Secondly, those HI genes for which the null mutant *is *fitness compromised are no more likely than the remainder of the HI gene set to be retained across the species (the *P *value across the yeast species for preferential retention = 0.3). Moreover, there is no significant correlation between the null mutant fitness for a given HI gene, and the fraction of species in which its orthologues are retained (correlation coefficient = 0.06).

### Haploinsufficiency in yeast is predictive

We also found that haploinsufficiency in yeast is a good predictor for the phenotype of orthologues in higher eukaryotes. Members of a set of 299 published human haploinsufficient genes, assembled via text mining (following the method described by Dang *et al. *[20]), are significantly more likely than other human genes to have a yeast orthologue that is HI (*P *< 10^-4^). A total of 83 of these human haploinsufficient genes have a BLAST best-hit orthologue in *S. cerevisiae *(consistent with the approximately 25% likelihood of a human gene being associated with a yeast orthologue by this method), and 40 of these (unique) orthologues are also HI in yeast.

We therefore defined a set of candidate human HI genes based upon the phenotype of their yeast orthologues (using unique yeast-human orthologue matches), listed in Additional file 2, and examined their gene ontology and disease associations. Of our 563 human gene candidates, 104 are associated in the Online Mendelian Inheritance in Man (OMIM) database [21] with a genetic disease (see Additional file 3). A GO enrichment search of the candidate HI/disease-associated set suggests an over-representation of gene functions essential in tumourigenesis and metastasis. DNA damage response and repair, apoptosis, and the oxidative stress response, along with cell migration and cell-cell adhesion terms are all enriched with a *P *value of less than 0.0005 (corrected for multiple testing).

Our predicted HI/OMIM-associated set includes the known HI tumour suppressor *NF1*, along with a cluster of four genes, *RPS19, RPL1, RPS7 *and *RPS24*, associated with Diamond-Blackfan anaemia (the latter three of which are novel HI predictions), and three connected with Williams syndrome: *RFC2, STX1A *and *PSPH *(the latter two being novel HI predictions). Since both of these conditions have independently been associated with haploinsufficiency [5,22], their presence among our predicted set provides encouraging support for our yeast-based method. Further clusters include four genes related to colorectal cancer (a GO enrichment *P *value of 0.0006). None of these four genes has previously been annotated as HI according to our text-mining search. In addition, we make novel HI predictions for several genes associated with *Xeroderma pigmentosum *and, by extension, defective nucleotide excision repair. Genes involved in the Kyoto Encyclopedia of Genes and Genomes (KEGG) pathways [23] of Huntingdon's disease, Parkinson's disease and Alzheimer's disease are also over-represented (*P *< 0.02 for all; see Additional file 3).

### Haploinsufficient genes are over-represented on the mating-type chromosomes of the Fungi

Aside from disease, in heterogametic eukaryotes, haploinsufficiency is obviously relevant with respect to the sex chromosomes since the dosage of the sex-linked genes can vary by a factor of two between the sexes. We previously noted [8] that chromosome III of *S. cerevisiae*, which carries the mating locus (*MAT*), is enriched for genes that are HI under different nutrient-limited conditions (*P *< 10^-50^). We argue that this accumulation was selectively advantageous upon its occurrence in an ancestral yeast, and has been subsequently retained across the lineage.

**Figure 1:**
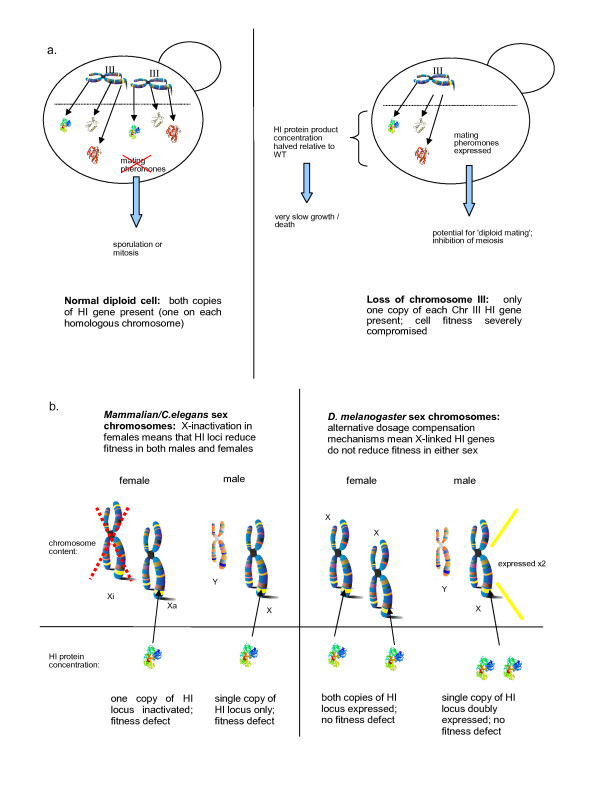
**Consequences of the accumulation of haploinsufficient genes on the mating-type chromosomes of fungi and the sex chromosomes of animals**. **(a) **In the diploid yeast cell, loss of one copy of the mating chromosome allows expression of the mating pheromones (typically suppressed in the diploid), generating a mating-competent diploid. The accumulation of haploinsufficient (HI) genes on this chromosome is proposed to be a selective mechanism against its loss, compromising fitness in the chromosome III monosome to such a degree as to preclude diploid mating. **(b) **In animals, the presence of HI genes on the sex chromosomes should incur a selective penalty in the heterogametic sex. In mammals and *Caenorhabditis elegans*, X chromosome inactivation in the female halves the expression of the genes along the entire chromosome, resulting in selective pressure against X-linked HI genes in the homogametic sex as well. In contrast, in *Drosphila melanogaster *males, the expression of the lone X chromosome is doubled, thus any HI genes present on the chromosome should present no detriment to the fitness of either sex.

As to the selective advantage, we have previously proposed that the accumulation of HI genes on this chromosome is a mechanism that leads to cells that have lost one copy of chromosome III being eliminated from the population. Generally, mating ability is repressed in diploid cells due to the presence of both mating-type alleles, but a diploid bearing only one copy of chromosome III can express the haploid mating programme, becoming a so-called 'diploid mater'. Such aneuploids would be unable to sporulate and would give rise to (fitness-compromised) triploid progeny on mating with a haploid partner (Figure 1a); thus, chromosome III monosomy is a serious detriment to the population.

Here, we have found that the over-representation of HI genes on *S. cerevisiae *chromosome III also holds when the phenotype was defined in turbidostat culture (*P *= 10^-5^). Thus the effect is not nutrient specific and holds true in yeast growing in near-optimal conditions. To test the loss-of-chromosome III hypothesis, we examined whether the observed *MAT *linkage also holds in other fungi having the same mating-type determination system. Indeed, orthologues of HI genes from *S. cerevisiae *chromosome III are found to be over-represented on the chromosomes bearing the mating-type locus in species throughout the Ascomycota (with *P *values ranging from 10^-2 ^to < 10^-19^). By constructing the relevant heterozygous deletion mutants, we have confirmed, in a subset of these *MAT*-linked HI orthologues, that the haploinsufficient phenotype does indeed carry over to two other yeast species. Mutants for 4 of 5 *Saccharomyces kluyveri *genes, and 9 of 10 deletions in a *Kluyveromyces lactis *diploid, grow significantly slower than the wild type, corresponding to *P *values of 7 × 10^-3 ^and 4 × 10^-6^, respectively (see Table 3). It should be noted the batch growth assays performed for the non-*cerevisiae *yeasts are significantly less sensitive than continuous culture competition experiments [8], and hence small (but significant) differences in growth rate will not be detected. That a growth defect (much less a significantly lower growth rate) is observed at all in batch culture is indicative of a strongly haploinsufficient phenotype.

**Table 3:** *Kluyveromyces lactis *and *Saccharomyces kluyveri *mating-type chromosome genes for which heterozygous deletion mutants were constructed, along with the measured relative maximum exponential growth rate

Species	Gene for which the strain is heterozygous	*Saccharomyces cerevisiae *orthologue (ORF name/gene name)	Relative maximum exponential growth rate (± 9%)
*Kluyveromyces lactis *CK11	-		1

*Kluyveromyces lactis*	KLLAC08910	YCR060W/*TAH1*	0.88

	KLLAC08778	YCR065W/*HCM1*	0.81

	KLLAC08723	YCR067C/*SED4*	0.87

	KLLAC02101	YCR044C/*PER1*	0.92

	KLLAC03520	YCR035C/*RRP43*	0.90

	KLLAC08569	YCR071C/*IMG2*	0.91

	KLLAC02145	YCR043C/uncharacterised	0.93

	KLLAC03454	YCR037C/*PHO87*	0.97

	KLLAC03498	YCR036W/*RBK1*	1.05

	KLLAC03569	YCR033W/*SNT1*	0.83

*Saccharomyces kluyveri *WT (CBS 3802)	-		1

*Saccharomyces kluyveri*	SAKL0C04466	YCR031C/*RPS14A*	0.90

	SAKL0C04334	YCR033W/*SNT1*	0.91

	SAKL0C04290	YCR035C/*RRP43*	0.93

	SAKL0C02354	YCR044C/*COS16*	1.02

	SAKL0C01276	YCL036W/*GFD2*	0.97

ORF = open reading frame; WT = wild type.

The antiquity of the relationship between HI genes and the *MAT *chromosome is further suggested by the fact that the association holds not only in diploid species, but also in predominantly haploid yeasts such as *K. lactis *and *Ashbya *(*Eremothecium*) *gossypii *(Table 4). Since the loss of any chromosome from the haploid genome is lethal, additional selective mechanisms against the loss of the *MAT *chromosome should be unnecessary. Thus, HI genes should not be significantly clustered near the *MAT *locus in such species unless the arrangement is inherited from a common ancestor of the diploid/haploid yeasts.

**Table 4:** Significance of the over/under-representation of orthologues of *Saccharomyces cerevisiae *haploinsufficient (HI) genes on the mating-type chromosomes of ascomycetes and the sex chromosomes of animals

Organism	Predominant lifecycle phase	Mating-type or sex determination system	Orthologues of *Saccharomyces cerevisiae *HI genes on the mating-type/sex chromosome(s)
			*P *value for over-representation

*Saccharomyces cerevisiae*	Diploid	Bipolar sexuality	<10^-50^

*Saccharomyces castellii*	Diploid	Bipolar sexuality	<10^-10^

*Candida glabrata*	Diploid	Bipolar sexuality	<10^-10^

*Saccharomyces kluyveri*	Diploid	Bipolar sexuality	<10^-12^

*Kluyveromyces lactis*	Haploid	Bipolar sexuality	<10^-19^

*Ashbya gossypii*	Haploid	Bipolar sexuality	0.02

*Candida albicans*	Diploid	Asexual	0.8

*Candida dubliniensis*	Diploid	Asexual	0.6

*Debaryomyces hansenii*	Haploid	Bipolar sexuality	0.05

*Yarrowia lipolytica*	Haploid	Bipolar sexuality	0.002

			*P *value for under-representation

*Homo sapiens*	Diploid	X/Y	0.02

*Pan troglodytes*	Diploid	X/Y	0.04

*Mus musculus*	Diploid	X/Y	0.05

*Rattus norvegicus*	Diploid	X/Y	0.04

*Equus caballus*	Diploid	X/Y	0.01

*Bos taurus*	Diploid	X/Y	0.3

*Canis familiaris*	Diploid	X/Y	0.02

*Gallus gallus*	Diploid	Z/W	0.3

*Drosophila melanogaster*	Diploid	X/Y	0.9

*Caenorhabditis elegans*	Diploid	X/0	0.02

The only species not exhibiting linkage of HI genes to *MAT *are the closely related pathogens *Candida albicans *and *Candida dubliniensis *(see Table 4). These species possess only a parasexual cycle and are believed to be incapable of undergoing meiosis [24], in contrast to the other Ascomycetes studied (including *Candida glabrata*, for which a putative sexual cycle has been observed; [25]).

### Orthologues of yeast HI genes are explicitly excluded from the sex chromosomes in higher eukaryotes

In the higher eukaryotes that have a heterogametic sex-determination system, the situation is the reverse of that in the Fungi. In the XY, X0, and ZW systems, all chromosomes are maintained in duplicate in both sexes, except for the sex chromosomes, for which there is only a single copy in one of the sexes. The haploid status of the sex chromosome means that the presence of HI genes on these chromosomes would detrimental to the fitness of the heterogametic sex. Indeed, we have found that the orthologues of yeast HI genes are significantly under-represented on the X chromosome of the human, mouse, rat, chimp, dog, cow and horse (XY species) and the X chromosome of the nematode worm (X0) (see Table 4). In general, the Y chromosome is too small, and carries too few yeast orthologues, to perform significance tests.

Genes on the X chromosome are also under-represented among the set of curated human haploinsufficient genes (6 of the 299 are on the X chromosome, a *P *value for under-representation of 0.005). Furthermore, 2 of the 10 HI orthologues (that is, genes in our predicted human haploinsufficient set) found on the human (and chimp) X chromosome (namely *NLGN4X *and *DDX3X*) are among the 12 human genes that retain a functional homologue on the Y chromosome (*P *= 0.003, hypergeometric test). This perhaps indicates selection for the maintenance of their two-copy status. The remaining 10 human X chromosome genes possessing a Y chromosome gametologue do not possess yeast orthologues.

## Discussion

It appears that there is a special relationship between yeast HI genes, or their orthologues, and the sex-determining or mating-type-determining chromosomes, which is conserved across the eukaryotes.

In *Saccharomyces cerevisiae*, haploinsufficient genes are over-represented on chromosome III (the chromosome bearing the mating-type locus). We found that, across the phylum Ascomycota, orthologues of *S. cerevisiae *HI genes are also represented on the chromosome that bears the mating-type locus. We propose that the linkage of HI genes to the mating-type loci represents an ancient arrangement that guards against the survival of diploid maters. These would be produced by the loss of a single copy of the mating-type chromosome and could lead to the generation of triploid hybrids, which would be detrimental to the fitness of the population.

It might be contended that this arrangement is merely a 'frozen accident' that confers no selective advantage on the organism. For instance, an ancestral arrangement of genes on the mating chromosome that happened to include an over-representation of HI genes has been preserved over evolutionary time due to a failure of that chromosome to become rearranged through translocation events with the other chromosomes in the genome. It is certainly true that among the *Saccharomyces *'sensu stricto' (those yeasts which can mate with *S. cerevisiae*, and with each other, to produce viable hybrids), no translocation events have occurred between chromosome III and any of the other chromosomes. The same is true of chromosomes I and XIII [26,27]. However, Gordon *et al. *[28] have determined that 32 translocation events have occurred during the evolution of the *Saccharomyces *'sensu stricto' from the common, immediate pre-WGD, ancestral yeast, and 2 of these have involved the mating-type chromosome. Moreover, if one aligns the chromosomes of the pre-WGD species *Ashbya *(*Eremothecium*) *gossypii *with those of *S. cerevisiae*, around 200 translocation events can be detected and there is no evidence for any under-representation of such events on the mating chromosome (*P *of under-representation = 0.99). The most parsimonious explanation is therefore that the over-representation of HI genes on this chromosome has been maintained by selection.

The arrangement has been preserved since the pre-WGD common yeast ancestor and, among the species for which genome sequences are available, is lost only in *Candida albicans *and *Candida dublinensis*, two species that do not have a natural sexual cycle. This may lead to a relaxation of the selective pressure for the accumulation of HI genes on the *MAT-*bearing chromosome. In addition, both *C. albicans *and *C. dubliniensis *exhibit a high degree of genomic instability, with frequent chromosomal rearrangement and aneuploidy in populations of both laboratory strains and clinical isolates [29]. Not only does this imply a very low probability of retaining an ancient chromosomal arrangement (and thus the ancient *MAT-*HI linkage) in the absence of sex-driven selective pressure, but the necessity of tolerating frequent aneuploidy within the population might drive selection against the accumulation of HI genes on any one chromosome. Moreover, loss of one copy of the *MAT-*bearing chromosome 5 of *C. albicans *is required for adaptation to utilise alternative carbon sources, and to drive the white/opaque transition [30]. Since opaque cells have a vastly higher mating efficiency [30], loss of the *MAT *chromosome should actually be favourable for sex in *C. albicans*, and thus selective pressure should act against the accumulation of HI genes on this chromosome. This is indeed what we have observed, with a *P *value of 0.04.

In animals, as for the Fungi, we propose that a relationship between haploinsufficiency and the mating-type chromosomes (in this case, an explicit exclusion of HI genes from the X) arose in a common ancestor, and, owing to the selective advantage (or, more precisely, protection against a selective disadvantage) conferred, has been retained throughout the lineage. HI depletion is one of the features preserved in the highly-conserved mammalian X chromosome lineage, but has also been retained on the long-diverged *Caenorhabditis elegans *X.

Again, the idea of a 'frozen accident' should be considered, this time with the X chromosome of a common animal ancestor being devoid of HI orthologues and that situation being preserved due to a lack of genetic exchange between X and the autosomes. It is true that, as with the *Saccharomyces *'sensu stricto' yeasts, very few translocations involving the X chromosome have occurred among the mammals (three between the human X and cow chromosomes 7, 15 and 29; one from human X to rat 12 and rabbit 3; and five from human X to possum 4 and 7; [31]). Between the more distantly-related species, however, chromosomal rearrangements involving genes on the X are frequent. For example, Kohn *et al. *[32] mapped orthologues of 300 human X-chromosome genes to multiple chromosomes in *Tetraodon nigroviridis *(spotted pufferfish) and *Danio rerio *(zebrafish). In the latter species, 13 different linkage groups bear at least 5 of the X chromosome orthologues. Reversing the mapping, the human X chromosome is composed of over 30 distinct synteny blocks, from at least 10 different chromosomes, with respect to *T. nigroviridis *[33]. These ancestral rearrangements would have provided ample opportunity for HI genes to be transferred onto the X chromosome, and suggests that there has been active selection against this.

In many of the higher eukaryotes, dosage compensation mechanisms exist to bring the expression levels of the female's X-linked genes in line with those of the male [34]. In mammals, this is achieved by X chromosome inactivation and an analogous situation occurs in *C. elegans*, with expression levels of the genes on both of the X chromosomes in the XX hermaphrodite being reduced by a factor of two [34]. X inactivation therefore amounts to an additional selective pressure against the accumulation of HI genes on the X chromosome, since their presence would be detrimental in both the heterogametic male, and the female with an inactivated X (Figure 1b).

In contrast, the opposite mechanism operates in *D. melanogaster*, with the assembly of a male-specific ribonucleoprotein complex (the dosage compensation complex or DCC) modifying the chromatin structure on the male's X chromosome, and acting to double the expression levels of all its genes [34]. Thus, the presence of HI genes on the X chromosome should not adversely affect male fitness and, indeed, we have found that *D. melanogaster *(along with *Gallus gallus*, see below) does not exhibit a depletion of HI orthologues on the X chromosome (Figure 1b).

Similarly, the failure to observe a significant under-representation of HI orthologues on the Z chromosome of the chicken may be due to a lack of global dosage compensation in the avian ZW system (see [35]). This would result in an absence of selective pressure, in the (homogametic) male, against the Z linkage of HI genes. However, local downregulation of the expression of small clusters of genes on the chicken Z in (ZZ) males has been reported [35], and we note that HI orthologues on this chromosome are 2.5 times more likely to be found in the regions of peak expression (comprising 5 of the 18 genes in these regions having yeast orthologues), as opposed to these 'silenced' regions along the chromosome.

Haploinsufficient genes are being increasingly recognised for their role in human disease, and our results suggest that the phenotype may have had a major impact on genome organization over a significant stretch evolutionary time. Haploinsufficient *S. cerevisiae *genes are retained across the eukaryotic lineage and, furthermore, the haploinsufficiency of orthologous loci is conserved from the Fungi to the animals. This striking result suggests that our *S. cerevisiae *results might be used to speed the discovery of novel HI genes in humans, perhaps by guiding a gene knockout study in either human cell lines or mice.

## Methods

### Orthology assignments and phenotypic analysis

Orthology assignments were made using the InParanoid algorithm [36], and compared with the results of a BLAST [37] reciprocal best-hits search. GO_slim annotations for *S. cerevisiae *genes were retrieved from the Gene Ontology Consortium [20]. GO enrichment searches were performed using the Babelomics 4 FatiGO tool [38]. Associations of the human candidate set with inherited diseases were obtained from the Online Mendelian Inheritance in Man (OMIM) database [19].

Paralogous WGD pairs for the Saccharomycetales were obtained from Byrne and Wolfe [39]. *S. cerevisiae *over-expression phenotypes were from Sopko *et al. *[40] and homozygous deletion phenotypes from Winzeler *et al. *[41]. The sequence of the human X chromosome and the pseudoautosomal regions were obtained from Ross *et al. *[42] and Graves *et al. *[43], respectively.

### Statistical testing

To assess the significance of HI gene conservation, the number of HI genes having orthologues in a given species, given the number of *S. cerevisiae *HI genes, was compared against the whole-genome conserved proportion using a χ^2 ^or Fisher exact test (depending on sample size), with the null hypothesis of identical distribution. To assess the significance of HI gene numbers on a given chromosome, we calculated the hypergeometric probability, given the number of genes on the chromosome, and the number of HI genes in the genome.

A similar approach was taken in order to determine the significance of the distribution of HI orthologues across the chromosomes. The number of HI orthologues on the particular chromosome, given the total number of orthologues on the chromosome, was compared against the total number of HI orthologues in the species. Fisher's exact test was used to determine significance, using the null hypothesis that the distribution of HI orthologues would not differ from that of orthologues in general. We applied a similar methodology to confirm that the distribution of HI orthologues did indeed differ from the set of non-HI orthologues (data not shown). All findings of significance were reiterated using a z test for difference of proportions. Where necessary, *P *values were corrected for multiple testing.

### Deletion mutant construction

Candidate *K. lactis *and *S. kluyveri *genes were those on their chromosomes bearing the mating-type locus, which have a reciprocal best-hit orthologue on *S. cerevisiae *chromosome III which showed a haploinsufficient phenotype in selections in turbidostat culture. Of these, 10 *K. lactis*, and 15 *S. kluyveri *genes (see Table 3) were selected for targeting.

Strains were cultured in YPD rich medium (1% yeast extract, 2% peptone, 2% glucose), and *Escherichia coli *strains in LB medium (1% tryptone, 0.5% yeast extract, 1% NaCl). The pFA6a-KanMX and pAG25-NatMX plasmids were harvested from, respectively, *E. coli *transformants pFA6 and pAG25 by miniprep and digested with *Not*I. The resulting *Kan*MX/*Nat*MX gene was purified using a QIAquick kit (Qiagen, West Sussex UK).

Though amenable to transformation using techniques and plasmids designed for *S. cerevisiae*, the efficiency of homologous recombination in both *K. lactis *and *S. kluyveri *is much lower than in *S. cerevisiae*. Efficient gene targeting therefore requires at least 500 bases of homology between the regions flanking the target gene, and the replacement cassette. Such cassettes, containing either the *Kan*MX or *Nat*MX [44] gene (conferring resistance to geneticin 418 or nourseothricin, respectively), were constructed using the two-step polymerase chain reaction (PCR) method described by Wach *et al. *[45] with modifications. For the first step, two standard PCR reactions were set up to amplify the 500-nucleotide (nt) regions immediately upstream (or downstream) of the target gene, with genomic *K. lactis *CK11 or *S. kluyveri *CBS 3082 DNA as a template. Oligonucleotides were designed so as to concatenate an additional 26 bases homologous to the 5' (or 3') end of the marker gene onto the upstream (or downstream) amplified region.

These approximately 550-base upstream and downstream fragments were then used as primers in a second PCR reaction. The Roche Expand Long Template PCR system (Roche Diagnostics, West Sussex UK) containing a mixture of Taq and Tgo DNA polymerases was used, a typical reaction containing 0.5 μl (approximately 50 ng) of each of the first PCR products, 0.3-0.5 μg of the *Not*I-digested plasmid. PCR was carried out in a reaction volume of 50 μl, containing 200 μM of each dNTP, 1.75 mM MgCl_2_, 50 mM Tris/HCl pH 9.2, 16 mM (NH_4_)SO_4_, and 0.75 μl of enzyme mix. A Bio-Rad Thermo-Cycler 1000 (Bio-Rad laboratories, Hertfordshire UK) was programmed to initially denature the samples at 92°C for 120 s, then perform 10 cycles of 10 s at 92°C, 30 s at 50°C, 120 s at 68°C, followed by 25 cycles of 15 s at 92°C, 30 s at 50°C, and 120 s at 68°C, with an additional 20 s elongation for each successive cycle [46].

The resulting PCR product was used to transform *K. lactis *CK11 or *S. kluyveri *CBS 3082 following the method of Hill *et al. *[47]. Cells were grown in rich medium to a density of approximately 10^7 ^cells/l, harvested by centrifugation, washed in 10 ml 100 μM lithium acetate, and finally resuspended in 1 ml of 100 μM lithium acetate. A total of 100 μl of cell suspension was added per 10 μl of PCR product; 240 μl of 50% (w/v) poly(ethylene glycol)-3000; 30 μl 1 M lithium acetate; and 25 μl 2 mg/ml single-stranded (ss)-DNA. The suspension was incubated at 30°C with shaking for 30 min; 43 μl of dimethylsulfoxide added; and subsequently heat shocked at 42°C for 10 min. Cells were incubated in YPD for 3 h at 30°C, and G418 and nourseothricin-resistant transformants selected on YPD containing 0.2 mg/ml G418 and 0.1 mg/ml clonNAT (Werner Bioagents, Jena Germany), respectively. Correct marker integration was confirmed by colony PCR amplification of both the marker gene, and of the remaining copy of the target locus.

### Growth rate measurements

Stationary-phase cultures were diluted 1:100 in YPD, and 200 μl transferred into 1 well of a 96-well microtitre plate. A BMG Fluostar Optima microplate reader (BMG Labtech, Aylesbury UK) was used to take 600 nm absorbance readings at 10-min intervals over a period of 24-35 h. A curve-fitting algorithm, implemented in R, calculated the maximum growth rate from the measured growth curve, and the median was taken across all replicates of the strain in the plate. This value was compared with the median for each of the other strains in the same microplate, with significant growth-rate variation identified using a two-tailed *t *test at 95% significance. Measurements were repeated at least five times for each strain. The standard deviation of the repeated measurements was calculated, and the maximum among all strains (9%) was taken as the uncertainty in each strain.

### Statistical testing

The *P *value for the overlap between the sets of haploinsufficient genes in *S. kluyveri *or *K. lactis *and *S. cerevisiae *was calculated by assuming that the frequency of haploinsufficiency within the genome of these two species was equal to that in *S. cerevisiae *(approximately 20% in YPD). A *P *value can be ascribed to each orthologue set individually. With the null hypothesis that haploinsufficiency in *S. cerevisiae *and *S. kluyveri/K. lactis *is independent, the probability that both members of a given orthologue pair are HI is *P *= 0.2, and if the gene is HI in all three species, *P *= 0.2^2 ^= 0.04. The probability of observing *n *orthologue pairs in which both genes are HI, out of *N *pairs tested, is then given by the binomial probability of *n *successes, and (*N-n*) failures, with probability of success = 0.2. Thus the *P *value for 9/10 *K. lactis/S. cerevisiae *pairs having both orthologues HI is 4 × 10^-6^, and for 4/5 *S. kluyveri/S. cerevisiae *pairs both being HI is 7 × 10^-3^.

## Authors' contributions

SGO conceived the study and designed the investigations. PP identified yeast HI genes and investigated their duplicates. MdC performed all other investigations, constructed the *K. lactis *and *S. kluyveri *heterozygotes, and determined their growth rates. All three authors participated in data interpretation. MdC and SGO wrote the paper, and all three authors were involved in its revision.

## Supplementary Material

Additional file 1 **Supplementary Table 1**. Table of those genes retained in duplicate in *Saccharomyces cerevisiae *following the whole-genome duplication, in which at least one of the members of the duplicate pair is haploinsufficient. Click here for file

Additional file 2 **Supplementary Table 2**. Table of the 563 human genes predicted, on the basis of the yeast orthologue phenotype, to be haploinsufficient. Genes are listed along with their gene ontology functional annotation, and that of the yeast orthologue. Click here for file

Additional file 3 **Supplementary Table 3**. Table of the 104 human genes predicted to be haploinsufficient, which are associated with an inherited disease in the Online Mendelian Inheritance in Man (OMIM) database. Genes are listed along with the related OMIM annotation and OMIM ID. Click here for file
